# Association of the angiotensinogen gene polymorphism with atherosclerosis and its risk traits in the Saudi population

**DOI:** 10.1186/1471-2261-13-17

**Published:** 2013-03-11

**Authors:** Mohammed Al-Najai, Paul Muiya, Asma I Tahir, Samar Elhawari, Daisy Gueco, Editha Andres, Nejat Mazhar, Nada Altassan, Maie Alshahid, Nduna Dzimiri

**Affiliations:** 1 Genetics Department, King Faisal Specialist Hospital and Research Centre, Riyadh, 11211, Saudi Arabia; 2 King Faisal Heart Institute, King Faisal Specialist Hospital and Research Centre, Riyadh, 11211, Saudi Arabia

**Keywords:** Angiotensinogen polymorphism, Primary hypertension, Type 2 diabetes mellitus, Obesity, Coronary artery disease, Gene-disease interactions, Pleiotropy

## Abstract

**Background:**

Angiotensinogen (AGT) constitutes a central component of the renin-angiotensin system that controls the systemic blood pressure and several other cardiovascular functions and may play an important role in atherosclerosis pathways. In this study, we employed TaqMan genotyping assays to evaluate the role of 8 *AGT* variants in primary hypertension (HTN), type 2 diabetes mellitus (T2DM), and obesity as a possible trigger of coronary artery disease (CAD) in a population of 4615 angiographed native Saudi individuals.

**Methods:**

Linkage analysis was done by using the Affymetrix Gene Chip array, sequencing by using the MegaBACE DNA analysis system and genotyping accomplished by TaqMan chemistry using the Applied Biosystem real-time Prism 7900HT Sequence Detection System.

**Results:**

Six variants, rs2067853 GG [Odds ratio(95% Confidence Interval) = 1.44(1.17-1.78); p = 0.001], rs7079 [1.49(1.20-1.85); p < 0.0001], rs699 G [1.19(1.08-1.13); p < 0.0001], rs3789679 A [1.51(1.14-1.99); p = 0.004], rs2148582 GG [1.31(1.11-1.55); p = 0.002] and rs5051 TC + CC [1.32(1.13-1.60); p = 0.001] conferred risk for HTN (3521 cases versus 1094 controls). The rs2067853 (p = 0.042), rs699G (p = 0.007) and rs5051 (p = 0.051) also conferred risk for myocardial infarction (MI; 2982 vs 1633), while rs3789679 A (p < 0.0001) and GA + AA (p < 0.0001) as well as rs4762G (p = 0.019) were associated with obesity (1576 vs 2458). However, while these variants appeared to be also associated with CAD (2323 vs 2292), only the rs7079G (p = 0.035) retained its significant relationship. Interestingly, among the haplotypes constructed from these SNPs, the baseline 8-mer haplotype, GGTGGGGT (χ^2^ = 7.02; p = 0.0081) and another GGCGGAGT (χ^2^ = 5.10; p = 0.024), together with several of their derivatives were associated with HTN. T2DM was associated with two 8-mer haplotypes, GGTAGGAC (χ^2^ = 5.66; p = 0.017) and ATTGAGAC (χ^2^ = 5.93; p = 0.015), obesity with GGCGGAGT (χ^2^ = 9.49; p = 0.0021) and MI was linked to ATTGGGAC (χ^2^ = 6.68; p = 0.010) and GGTGGGAT (χ^2^ = 4.25; p = 0.039). Furthermore, several causative haplotypes were also shared among the risk traits as well as with CAD.

**Conclusion:**

These results point to *AGT* as independently conferring risk for various cardiovascular traits, and possibly interacting with these traits in events leading to atherosclerosis.

## Background

The renin-angiotensin-aldosterone system (RAAS) is the core regulator of systemic blood pressure. An essential component of this system is angiotensinogen (AGT), a serum renin glycoprotein substrate synthesized and released in the liver as the precursor of angiotensin, the hormone that constitutes part of the systemic blood pressure control system [1]. By virtue of the function of AGT in regulating blood pressure, changes in its gene sequence are likely to play an important role in the pathogenesis of cardiovascular risk traits, such as hypertension, as well as manifestation of coronary artery disease (CAD) [2,3]. Several AGT variants, including the rs4762 (p.M207T, also formerly known as p.T174M) and rs699 (p.M268T, also referred to as p.M235T), have been associated with susceptibility to various cardiovascular risk traits, such as primary hypertension (HTN) [4-10] and type 2 diabetes (T2DM) [11,12], that are linked to the development of CAD. These variants have similarly been implicated not only in the manifestation of CAD [13-23], but also in the severity of atherosclerosis [24,25]. The fact that the *AGT* gene constitutes a risk for these disease traits as well as CAD strongly implicates gene-disease interactions in pathways that trigger atherosclerosis, a subject that has attracted considerable research interest recently. Indeed, a number of studies have indicated that such gene-trait or gene-gene interactions contribute to events leading to complex disorders, such as CAD [5,26-31]. However, this subject has not been exhaustively addressed yet. Besides, the notion of some of the *AGT* variants or their interactions with such disease risk traits playing a role in CAD/myocardial infarction (MI) has been refuted by a number of studies in various ethnic groups [21,22]. We recently described associations of several *AGT* variants with CAD in heterozygous familial hypercholesterolaemia through linkage and population-based association studies in a large, angiographed and ethnically homogeneous Saudi cohort [9]. In the present study, we specifically asked the question whether alterations in the *AGT* gene are a common cause for different cardiovascular risk factors, thereby posing an additional risk to acquiring CAD in individuals harbouring such risk traits. We employed the same study population previously engaged in the early onset CAD study.

## Methods

### Study population

The initial linkage study was performed in a Saudi family of eleven with heterozygous familial hypercholesterolaemia, in which the primary proband underwent triple bypass surgery at the age of 14 years (Additional file 1: Family Suppl data). This was followed by a case–control study in a total of 4615 native Saudi individuals from all five regions of the country, comprising 850 (18.4%) from the Eastern, 1008 (21.8%) from the Central, 1207(26.2%) from the Northern, 1184 (25.7%) from the Southern and 366 (7.7%) from the Western Region. This population consisted of 2323 CAD patients (1777 males and 546 females, mean age 60.2 ± 0.2 yr) with angiographically determined narrowing of the coronary vessels by at least 50% and 2292 angiographed controls (1245 male and 1047 female, mean age 50.6 ± 0.4 yr). Among these, 3521 individuals had primary (essential) hypertension (HTN) (Table 1), defined as ≥140 mm Hg systolic blood pressure or ≥90 mm Hg diastolic pressure based on The Sixth Report of the Joint National Committee on Prevention, Detection, Evaluation, and Treatment of High Blood Pressure (JNC VI) criteria [32]. Accordingly, essential, primary or idiopathic hypertension is defined as high blood pressure (HBP) in which secondary causes such as renovascular disease, renal failure, pheochromocytoma, aldosteronism, or other causes of secondary hypertension or Mendelian forms (monogenic) are not present [32]. The third subset of interest comprised 2544 individuals with T2DM (formerly known as non-insulin-dependent diabetes mellitus or adult onset diabetes). The National Diabetes Data Group of the USA and the second World Health Organization Expert Committee on Diabetes Mellitus (1998) [33] define type 2 diabetes mellitus as a metabolic disorder that is characterized by high blood glucose (generally described as fasting glucose level >126 mg/dL) in the context of insulin resistance and its relative deficiency. The fourth group constituted 1576 obese patients with body-mass index of ≥30.0 kg/m^2^, and classified as the obesity subset. Furthermore, 2982 individuals had established MI. Among these subsets of patients, some individuals harboured a combination of two or possibly three of the cardiovascular risk traits. The overall exclusion criteria for the disease cases were major cardiac rhythm disturbances, incapacitating or life-threatening illness, major psychiatric illness or substance abuse, history of cerebral vascular disease, neurological disorder, and administration of psychotropic medication. The controls (CON) for CAD were a group of individuals undergoing surgery for heart valvular diseases, and those who may have reported with chest pain, but were established to have no significant coronary stenosis by angiography. All patients included in study were undergoing their standard prescribed treatments for their respective ailments at our Institution. These included Zocor Pravastatin and Lipostat for hyperlipidaemia as well as Renitec, Hyzaar, Capoten, atenolol, metoprolol and carvedil for hypertension. Exclusion criteria for this group were among others diseases such as cancer, autoimmune disease, or any other disorders likely to interfere with variables under investigation. This study was performed in accordance with the regulations laid down by the King Faisal Specialist Hospital and Research Centre Ethics Committee in compliance with the Helsinki Declaration (http://www.wma.net/en/30publications/10policies/b3/index.html) and all participants signed an informed consent.

**Table 1:** Important clinical features and demographics of the studied individuals

	**Controls**			**Cases**		
	**All**	**Male**	**Female**	**All**	**Male**	**Female**
**CAD**	2292	1245(0.54)	1047(0.46)	2323	1777(0.76)	546(0.24)
**MI**	853	558(0.65)	295(0.35)	2180	1682(0.77)	498(0.23)
**T2DM**	954	524(0.55)	430(0.45)	1589	1175(0.74)	414(0.26)
**HTN**	1559	828(0.53)	731(0.47)	1962	1474(0.75)	488(0.25)
**hChol**	593	312(0.53)	281(0.47)	1078	796(0.74)	282(0.26)
**lHDL**	729	487(0.67)	242(0.33)	1181	979(0.83)	202(0.17)
**hTG**	427	267(0.63)	160(0.37)	728	554(0.76)	174(0.24)
**hLDL**	886	456(0.51)	430(0.49)	965	717(0.74)	248(0.26)
**FH**	542	299(0.55)	243(0.45)	359	287(0.80)	72(0.20)
**OBS**	888	385((0.43)	503(0.57)	857	573(0.67)	284(0.33)
**Smokers**	685	639(0.93)	46(0.07)	1059	1031(0.97)	28(0.03)
**VD** 1	0	0	0	885	642(0.73)	243(0.27)
2	0	0	0	456	356(0.78)	100(0.22)
3	0	0	0	965	717(0.74)	248(0.26)
**Clinical characteristics**						
**Age**	50.6 ± 0.4	51.2 ± 0.5	49.8 ± 0.5	60.3 ±0.2	59.8 ± 0.3	61.8 ± 0.54
**BMI**	29.0 ±0.2	27.97 ± 0.2	30.3 ± 0.3	28.9 ± 0.1	28.3 ± 0.1	31.0 ± 0.3
**Total Chol**	4.51 ± 0.02	4.42 ± 0.03	4.62 ± 0.03	4.48 ± 0.02	4.43 ±0.03	4.66 ± 0.05
**HDL-Chol**	1.26 ±0.01	1.18 ±0.03	1.33 ±0.01	1.15 ±0.01	1.15 ± 0.01	1.25 ± 0.02
**LDL-Chol**	2.76 ± 0.02	2.73 ± 0.03	2.80 ±0.03	2.71 ±0.02	2.68 ± 0.02	2.84 ±0.06
**TG**	1.52 ± 0.02	1.60 ± 0.03	1.44 ± 0.03	1.78 ± 0.02	1.78 ± 0.03	1.78 ± 0.05
**Fasting glucose**	6.87 ± 0.16	6.80 ± 0.23	6.92 ± 0.22	9.45 ± 0.31	9.27 ± 0.37	9.88 ± 0.56
**BP**	120/83	119/81	121/82	128/84	130/85	127/83

The table shows the important clinical features of the studied coronary artery disease patients versus angiographed controls, as well as the portion of disease traits in these groups. The numbers in brackets give the proportions of the total (all) values. MI, myocardial infarction; T2DM, type 2 diabetes mellitus; VD, number of diseased vessels; FH, family history of CAD; hChol, hypercholesterolaemia; lHDL, low high density lipoprotein; hTG, high triglycerides; hLDL, high low density lipoprotein cholesterol; HTN, BP as mm HG, hypertension, BP, blood pressure. Lipid and glucose levels are given as mmol/L.

#### Linkage analysis and screening for mutations

Five ml of peripheral blood was sampled in EDTA tubes from each of the study individuals after obtaining their written consent, and genomic DNA extracted from leukocytes by the standard salt method using PUREGENE DNA isolation kit (Qiagen Sciences, Germantown, MD, USA). For the genome-wide scanning with the Affymetrix Gene Chip 250 sty1 mapping array (Affymetrix Inc., Santa Clara, CA, USA), 250 ng of genomic DNA was digested with the restriction endonuclease StyI, mixed with Sty1 adaptors and ligated with T4 DNA ligase. The mixture was added to four separate PCR reactions, amplified, pooled and purified to remove the unincorporated dNTPs. The PCR product was then fragmented, biotinylated, hybridized to the 250 sty1 array for 18 h, washed, stained and scanned as recommended by the manufacturer. SNP genotypes, linear chromosomal locations and marker ordering were accomplished using the Affymetrix GeneChip® Genotyping Analysis Software (GTYPE) Version 4.0. Multipoint parametric linkage analysis was performed using the GeneHunter Easy Linkage analysis software 4.0 (Affymetrix, Inc., Santa Clara, CA, USA) for estimating the LOD scores. The disease was assumed to be an autosomal dominant trait with 100% penetrance. Copy Number Analyzer for GeneChip® (CNAG) Ver. 3.0 (Affymetrix, Inc., Santa Clara, CA, USA) was employed to check the shared chromosomal regions of homozygosity.

#### Sequencing of the gene

The linkage study was followed by sequencing exons and intron-exon junctions of the *AGT* gene in the family members and 200 individuals from the general population using the MegaBACE DNA analysis system (Amersham Biosciences, Sunnyvale, CA, USA). Briefly, the DNA was subjected to PCR by standard methods described elsewhere. Five μl of PCR product were then treated with 2 μl of ExoSAP-IT (USB Corporation, Cleveland, Ohio, USA) at 37°C for 30 min to allow the hydrolytic removal of excess primers and dNTPs by Exonuclease 1 and Shrimp Alkaline phosphatase. The enzymes were inactivated at 80°C for 15 min, and the sequencing reaction was initiated by mixing 2 μl DNA, 1 μl of 5 μmol primer, 8 μl of DYEnmic ET Dye Terminator (Amersham Biosciences, Buckinghamshire, UK) and 9 μl of distilled water. The mixture was thermally cycled 40x at 95°C for 20 sec, 50°C for 15 sec, and 60°C for 1 min. Unincorporated dye-labelled terminators were removed by gel-filtration through the DyeEx 96 plate (Qiagen, GmbH, Hilden, Germany). The eluent was vacuum-dried and dissolved in 10 μl of loading solution (GE Healthcare UK Ltd, Little Chalfont, Buckinghamshire UK) for sequencing. Data were analyzed for SNPs by Lasergene software (DNASTAR, Inc. Madison, WI USA).

#### Association experiments

Once the SNPs of interest were identified, genotyping was achieved by TaqMan chemistry using the Applied Biosystems real-time Prism 7900HT Sequence Detection System (ABI Inc. CA, USA). Primers and the TaqMan fluorogenic probes bearing a suitable reporter dye on the 5’-end and a quencher dye on the 3’-end were designed using the Primer Express software V2.0 (ABI Inc., Foster City, CA, USA) and procured from Applied Biosystems (ABI, Warrington, UK). One probe (for allele 1) was labeled with VIC dye and the other (for allele 2) with FAM dye at the 5’-end, and serial dilutions were run to determine the optimal working concentrations. For each reaction, a 25 μl mixture was prepared by mixing 5 μl containing 50 ng DNA, 12.5 μl of 2x Universal mix (Eurogentec, Liege Science Park, Seraing Belgium), 1.25 μl of 20x probe assay mix and 6.25 μl DNase-free distilled water. Three no-template controls were included in each plate for normalization of emission signal. The thermal profile for amplification for the first cycle occurred at 50°C for 2 min, and 95°C for 10 min, followed by 40 cycles of 94°C for 15 sec, and 60 C for 30 sec. The plates were then scanned for FRET signal using the 7900HT sequence detection system and data analyzed using SDS 2.0 software (ABI, Foster City, CA, USA).

#### Statistical analysis

Comparison of genotypes and alleles between different groups for continuous dependent variables was achieved by Analysis of Variance (ANOVA) or Student’s test as appropriate. Categorical variables were analyzed by Chi-Square test, and logistic regression analysis was used to compute odds ratios and their 95% confidence intervals. Multinomial logistic regression was carried out to access the relationship of the SNPs with CAD in the presence of the different disease traits, and Bonferroni tests were carried out to correct for the effect of age on these relationships. All of these statistical analyses were performed using the SPSS software version 20 (SPSS Inc., Chicago, USA). The haplo.stats package in the R Statistical Computing software (http://www.r-project.org/) was used to perform haplotype-based association analysis (http://mayoresearch.mayo.edu/mayo/research/schaid_lab/software.cfm). Odd ratios for haplotypes were calculated using the baseline haplotype GGTGGGGT as reference, and the Haplotype Score statistic for the association of a haplotype with the binary trait calculated as in Schaid *et al*. (2002) [34] and Lake *et al*., (2003) [35]. Significance of association was determined between haplotypes and the case–control status - a binomial trait denoting whether or not a patient had the disease. Data are expressed as mean ± SEM and associations with a two-tailed *p* value <0.05 was considered statistically significant.

## Results

### Genotyping and disease

In the present study, we focused on the possibility that the *AGT* gene confers risk for cardiovascular risk traits in a fashion that may influence pathways to acquiring CAD, following an initial study linking the gene to early onset of CAD in heterozygous familial hypercholesterolaemia. Interrogation of our sequencing data in the family members and 200 individuals from the general population with the HapMap (Http://hapmap.org) and Perlegen (http://genome.perlegen.com) databases led to the identification of several known and unfamiliar SNPs within the coding as well as the 3’ and 5’ untranslated regions of the AGT gene. This resulted in the selection of a total of 8 SNPs as informative for further evaluation. Apart from their frequencies in the study family and general population, selection of these SNPs was also partly based on available information on their impact on disease, to allow comparison with other data in the literature.

### Association of AGT polymorphism and cardiovascular disease traits

**Figure 1:**
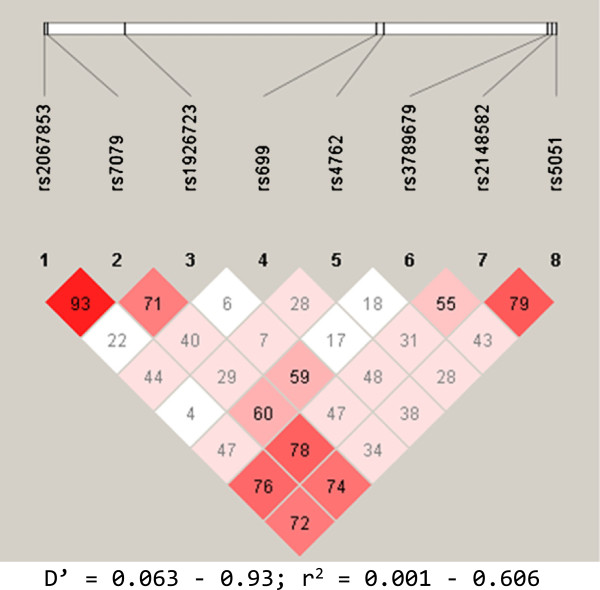
**Linkage disequilibrium structure of the eight studied angiotensinogen SNPs.** The SNPs are shown sequentially as they appear on the chromosome (not to scale). D’ = coefficient of linkage disequilibrium; r = regression coefficient of linkage disequilibrium.

The SNPs selected for the study included (1) rs2067853G > A, (2) rs7079G > T, (3) rs1926723T > C (4) rs699G > A, (5) rs4762G > A, (6) rs3789679G > A, (7) rs2148582 G > A and (8) rs5051T > C, sequentially numbered by the order of their chromosomal/genomic locations (Figure 1), and further referred to by these numbers for easy readability. Two of the SNPs (rs699 and rs4762 were missense mutations, while the rest were either intronic or resided in the untranslated regions of the gene (Additional file 2: Suppl data 1). The analysis for HTN (3521 cases versus 1094 controls) demonstrated causative associations for six of the studied variants with the disease. Thereby, both the rs2067853 G [Odds ratio(95% Confidence Interval) = 1.15(1.04 - 1.28); p = 0.007] and the recessive rs2067853 GG [1.44(1.17 - 1.78); p = 0.001], rs7079 GG [1.49(1.20 - 1.85) p < 0.0001], rs699 G [1.19(1.08 - 1.31); p < 0.0001], rs699 (AG + GG) [1.26(1.10 - 1.46); p = 0.001], the rs3789679 A [1.51(1.14 - 1.99); p = 0.004], rs2148582 GG [1.31(1.11 - 1.55); p = 0.002] and rs5051 (TC + CC) [1.32(1.13 - 1.60); p = 0.001] conferred risk for HTN (Table 2). Our data also implicated three of these variants, the rs2067853 GG [1.16(1.01 - 1.33); p = 0.042] as well as the rs699 G [1.13(1.03 - 1.23); p = 0.007] and rs699 AG + GG [1.20(1.09 - 1.31); p < 0.0001] in MI, while two others, rs7079 GG [1.15(1.00 - 1.33); p = 0.054] and rs5051 (TC + CC) [1.12(1.00 - 1.25); p = 0.051] displayed borderline causative properties. Furthermore, obesity was linked to both the rs3789679 A allele [1.59(1.28 - 1.98); p < 0.0001] and its combined GA + AA genotypes [1.57(1.24 - 2.00); p < 0.0001] as well as the rs4762 G [1.19(1.03 - 1.37); p = 0.019] (Table 2). Thus, these results point to common *AGT* variants as causative for hypertension, myocardial infarction and obesity in our population. Apart from the rs1926723T > C (p = 0.074) which was only very weakly implicated in T2DM, none of the other studied variants showed any definable relationship with this disease in the general population. Besides, some differences were observed in the distribution of the causative alleles by gender, whereby the rs699 G (p = 0,014), rs4762 G (p = 0.019) and rs5051 C (p = 0.04) were predominantly found among the male, while the rs2148582 G (p = 0.061) and rs1926723 T (p = 0.074) were weakly related to the female gender. However these differences did not seem to have any impact on the relationships of the gene variants with disease.

**Table 2:** **Association of the *AGT *gene variants with cardiovascular risk traits**

**Variant**	**Allele/Genotype**	**CON**	**Cases**	**O.R.(95%C.I.)**	**P-value**
**Hypertension**		**N = 1596**	**N = 3521**		
rs2067853G > A	G	0.681	0.711	1.15(1.04-1.28)	0.007*
	GG	0.870	0.906	1.44(1.17-1.78)	0.001**
rs7079G > T	G	0.683	0.712	1.14(1.03-1.27)	0.012
	GG	0.879	0.915	1.49(1.20-1.85)	<0.0001**
rs699G > A	G	0.560	0.602	1.19(1.08-1.31)	<0.0001**
(p.M268T)	GG	0.791	0.821	1.21(1.03-1.44)	0.024
	AG + GG	0.328	0.383	1.26(1.10-1.46)	0.001**
rs3789679G > A	A	0.028	0.042	1.51(1.14-1.99)	0.004*
	AA	0.005	0.012	2.70(1.07-6.83)	0.036
	GA + AA	0.052	0.072	1.41(1.05-1.90)	0.022
rs2148582G > A	G	0.563	0.587	1.10(1.00-1.21)	0.051
	GG	0.786	0.828	1.31(1.11-1.55)	0.002*
rs5051T > C	C	0.571	0.599	1.12(1.02-1.24)	0.02
	TC + CC	0.800	0.843	1.32(1.13-1.60)	0.001**
**Myocardial infarction**		N = 1584	N = 3033		
rs2067853G > A	GG	0.889	0.902	1.16(1.01-1.33)	0.042
rs7079G > T	GG	0.898	0.911	1.15(1.00-1.33)	0.054
rs1926723T > C	T	0.961	0.969	1.25(0.99-1.57)	0.063
rs699G > A	G	0.573	0.602	1.13(1.03-1.23)	0.007*
	GG	0.803	0.820	1.20(1.00-1.25)	0.047
	AG + GG	0.343	0.384	1.20(1.09-1.31)	<0.0001**
rs5051T > C	TC + CC	0.822	0.838	1.12(1.00-1.25)	0.051
**Obesity**		N = 3342	N = 1721		
rs4762G > A	G	0.887	0.903	1.19(1.03-1.37)	0.019
(p.M207T)	AG + GG	0.192	0.167	0.85(0.72-0.99)	0.040
rs3789679G > A	A	0.032	0.050	1.59(1.28-1.98)	<0.0001**
	AA	0.008	0.015	2.00(1.12-3.58)	0.019
	GA + AA	0.056	0.085	1.57(1.24-2.00)	<0.0001**

The table compares the AGT allele/genotype frequencies (as proportions), odd ratios (O.R.), 95% confidence levels (95% C. I.) and the significance levels of the associations in hypertension, myocardial infarction and obesity versus their respective angiographed controls. The letter N represents the population size. *P < 0.01, **P < 0.001 by χ^2^ test.

### Test for interactive gene-traits relationship on coronary artery disease

In order to test the likelihood of gene interactions with risk traits as an underlying cause for CAD in our studied population, multinomial logistic regression was performed to access the relationship of the SNPs with CAD in the presence of the different disease traits (Additional file 2: Suppl data 3–8). To begin with, apart from the major traits themselves, myocardial infarction, hypertension, type 2 diabetes mellitus and obesity, only the rs7079 T appeared to retain its causative association with CAD following the Bonferroni adjustment (Tables 3 and 4; Additional file 2: Suppl. data 9). Multinomial regression analysis indicated that this association was also retained in the presence of MI. The rs1926723 C was similarly associated with CAD in the presence of MI, while rs2148582A also became significantly associated in presence of HTN (0.002) and T2DM (p = 0.019). On the other hand, the rs3789679 A was protective against CAD in presence of MI (p = 0.001), HTN (p = 0.002), T2DM (p = 0.019). Thus, the results demonstrate that the relationships of these variants are retained in individuals harbouring CAD, pointing to their independence from the presence or absence of the later. Put together, the impact of the *AGT* variants on CAD seemed to depend on the prevailing disease condition.

**Table 3:** The relationship of the angiotensinogen variants with coronary artery disease adjusted for the presence of its risk factors

**Variable**	**Estimate**	**S. E**	**t**	**P-value**	**95%C.I**	
					**Lower**	**Upper**
Sex	−0.100	0.009	−11.296	<0.0001***	−0.122	−0.086
MI	0.555	0.010	58.143	<0.0001***	0.574	0.613
HTN	0.036	0.010	3.509	<0.0001***	0.009	0.051
T2DM	0.087	0.009	9.732	<0.0001***	0.063	0.100
OBS	−0.022	0.009	−2.435	0.015	−0.037	−0.003
Age	0.003	0.000	8.079	<0.0001***	0.002	0.003
FH	<0.00001	0.011	0.998	0.998	−0.021	0.021
rs7079T	−0.020	0.009	−2.163	0.031	−0.035	0.001
MI _Intercept	1.255	0.395	3.174	0.002*	0.480	2.030
MI * rs3789679A	−0.104	0.032	−3.229	0.001**	−0.168	−0.041
MI * rs1926723C	0.092	0.035	2.597	0.009	0.022	0.161
MI * rs7079G	0.027	0.012	2.345	0.019	0.004	0.050
HTN _Intercept	0.371	0.549	0.675	0.500	−0.706	1.447
HTN* rs2148582A	0.065	0.021	3.095	0.002*	0.024	0.107
HTN * rs3789679A	−0.074	0.035	−2.099	0.036	−0.143	−0.005
T2DM_ Intercept	−0.089	0.418	−0.212	0.832	−0.908	0.731
T2DM * rs2148582A	0.056	0.024	2.347	0.019	0.009	0.103
T2DM * rs3789679A	−0.099	0.044	−2.241	0.025	−0.186	−0.012
Gender_ Intercept	−0.342	0.487	−0.702	0.482	−1.298	0.613
Female * rs2148582A	0.103	0.030	3.405	0.001**	0.044	0.162
Male * rs2148582G	1.256	0.645	1.948	0.051	−0.008	2.520

The table displays the significant relationship of the angiotensinogen gene variants with coronary artery disease in presence of the various disease traits. Detailed analyses for the individual traits are given in the Additional file 1: Supplementary data file. S.E., standard error; t, Standard t-test MI, myocardial infarction; HTN, hypertension; T2DM, type 2 diabetes mellitus. OBS, obesity; FH, family history of coronary artery disease. *P < 0.01, **P < 0.0.001, ***P < 0.0001.

**Table 4:** Adjustment for age in the association of AGT variants with coronary artery disease

**Parameter**	**Estimate**	**S. E.**	**t**	**P-value**	**Adjusted P-value**	**95% C. I.**	
						**Lower**	**Upper**
A ≤44 * rs2148582A	−0.694	0.341	−2.038	0.042*	0.168	−1.362	−0.026
A ≤63 * rs5051C	−0.075	0.036	−2.05	0.04*	0.2	−0.146	−0.003
A ≤68 * rs5051C	−0.119	0.04	−2.948	0.003***	**0.015**	−0.197	−0.04
A ≤51 * rs3789679A	−0.142	0.068	−2.068	0.039*	0.195	−0.276	−0.007
A ≤63 * rs3789679A	−0.138	0.064	−2.167	0.03*	0.15	−0.263	−0.013
A ≤68 * rs4762A	0.081	0.038	2.16	0.031*	0.155	0.008	0.155
A ≤51 * rs2067853A	−0.065	0.031	−2.106	0.035*	0.175	−0.126	−0.004
A ≤51 * rs7079G	0.066	0.025	2.687	0.007**	**0.035**	0.018	0.114

The table shows the Bonferroni adjustments for the influence of age on the significance of the relationship between the SNPs and coronary artery disease. The data shows that the significance for association of the two variants rs5051C (for ages 63–68) and rs7079 (44–51) with coronary artery disease was retained following Bonferroni correction. *P < 0.05, **P < 0.01, ***P < 0.005., age; S.E. standard error, t, standard t test C.I., confidence interval.

### Haplotype analysis

Haplotyping involving different combinations of the eight studied SNPs was performed for the disease traits, employing the most prevalent 8-mer sequence GGTGGGGT (frequency = 0.338) as the baseline for comparative analysis of their relationships. To begin with, it was noteworthy that this baseline haplotype itself conferred risk for HTN (χ^2^ = 7.02; p = 0.0081). As shown in Table 5, its 7-mer derivatives, GGTGGGG (block 1–7; χ^2^ = 10.23; p = 0.0014) constructed from SNPs 1–7 as well as GTGGGGT (block 2–8; χ^2^ = 7.03; p = 0.008) constructed from SNPs 2–8, were also significantly associated with HTN. The trend in these associations trickled down to the shorter sequences with the highest level of significance being exhibited by its 4-mer GGTG (χ^2^ = 13.23; p = 0.0003). Apart from the GGTGGGGT, a second 8-mer GGCGGAGT (χ^2^ = 5.10; p = 0.024) also constituted a risk for HTN, with its shorter derivatives displaying similar trends. Its 4-mer derivative GAGT (χ^2^ = 11.40; p = 0.0007 as well as its 3-mer AGT (χ^2^ = 13.77; p = 0.0002) exhibited the highest level of significance (Table 5). Besides, two other 8-mer haplotypes GGTAGGGT (χ^2^ = 9.87; p = 0.0017) and ATTGGGAC (χ^2^ = 6.75; p = 0.009) were protective against acquiring HTN (AGT Haplo Additional file 2: Suppl data). Similarly, their shorter variants displayed identical properties with, for example, the 5-mer TAGGG (block 3–7; χ^2^ = 12.70; p = 0.0004) showing the most significant characteristics. Further in-depth analysis of these trends reveals a number of interesting features. The most important of these is the observation that the primary 8-mer haplotypes displaying opposite characteristics differ only at the fourth nucleotide position, involving the rs699G > A. Tracking the shorter haplotypes down to the 3-mers (e. g. block 4–6; AGG versus GGA) indicates clearly that G allele of this SNP is consistently associated with causative effects, while its complementary A allele consistently mediates protective actions, therefore positioning this SNP at the core of the genomic link to HTN. Equally interesting changes were linked with nucleotides at positions 5 (rs4762G > A) and 6 (rs3789679G > A), indicating that the G allele was causative while the A allele was protective, and therefore pointing to these loci as a central component linking AGT with HTN.

**Table 5:** Association of selected angiotensinogen haplotypes with cardiovascular disease traits

**SNP Block**	**Haplotype**	**Pooled**	**Case**	**Control**	**χ**^**2**^	**P-value**
**Hypertension**						
1-8	GGTGGGGT	0.338	0.345	0.314	7.02	0.0081
1-7	GGTGGGG	0.355	0.365	0.327	10.23	0.0014*
2-8	GTGGGGT	0.339	0.347	0.316	7.03	0.008
1-5	GGTGG	0.408	0.417	0.381	8.97	0.0028*
4-8	GGAGT	0.022	0.025	0.013	11.48	0.0007**
4-7	GGAG	0.024	0.027	0.014	13.36	0.0003**
3-7	TGGGG	0.374	0.383	0.347	9.38	0.0022*
1-4	GGTG	0.465	0.475	0.431	13.23	0.0003**
5-8	GAGT	0.026	0.029	0.016	11.40	0.0007**
6-8	AGT	0.028	0.032	0.017	13.77	0.0002**
4-6	GGA	0.026	0.029	0.015	12.50	0.0004**
**Myocardial Infarction**						
1-8	ATTGGGAC	0.057	0.061	0.048	6.68	0.010
1-7	ATTGGGA	0.060	0.065	0.049	8.89	0.0029*
2-8	TTGGGAC	0.059	0.065	0.049	8.68	0.0032*
1-6	ATTGGG	0.077	0.083	0.064	10.72	0.0011**
3-8	TGGGAC	0.094	0.101	0.080	10.71	0.0011*
2-7	TTGGGA	0.063	0.069	0.051	11.40	0.0007*
1-5	ATTGG	0.077	0.083	0.064	11.22	0.0008*
4-8	GGGAC	0.093	0.100	0.080	10.17	0.0014*
1-4	ATTG	0.093	0.101	0.077	13.92	0.0002*
2-6	TTGGG	0.084	0.090	0.073	8.05	0.0046*
2-5	TTGG	0.086	0.092	0.074	8.26	0.0041**
**Obesity**						
1-7	GGCGGAG	0.015	0.021	0.012	10.08	0.0015**
2-8	GCGGAGT	0.014	0.019	0.011	9.22	0.0024**
1-6	GGCGGA	0.015	0.020	0.012	8.93	0.0028**
3-8	CGGAGT	0.014	0.019	0.011	8.73	0.0031**
4-8	GGAGT	0.022	0.029	0.018	10.96	0.0009**
3-7	CGGAG	0.015	0.020	0.012	9.03	0.0027**
5-8	GAGT	0.026	0.033	0.022	9.95	0.0016*
6-8	AGT	0.028	0.035	0.024	9.62	0.0019*
4-6	GGA	0.026	0.034	0.021	15.94	0.00005**

The most frequent 8-mer haplotype GGTGGGGT (0.338) was employed as the baseline to determine the relative effects of the other haplotypes. Number 1 denotes rs2067853, 2 is rs7079, 3 is rs1926723, 4 is rs699, 5 is rs4762, 6 is rs3789679, 7 is rs2148582 and 8 is rs5051 arranged sequentially by their chromosomal positions, and blocks represent the range of variants constituting the respective haplotypes. *P < 0.005, **P < 0.001 by χ^2^ test.

The analysis for T2DM also produced two causative 8-mer haplotypes, GGTAGGAC (χ^2^ = 5.66; p = 0.017) and ATTGAGAC (χ^2^ = 5.93; p = 0.015), as well as protective ATTAGGAC (χ^2^ = 4.40; p = 0.036). Similar trends were observed that culminated inversely with the size of the derivatives, whereby the association for the 6-mer ATTGAG (block 1–6; χ^2^ = 8.17; p = 0.004) was the most conspicuous (AGT Haplo Additional file 2: Suppl data). As in HTN, a couple of noticeable features could be identified pointing to the rs699G > A and rs4762G > A as distinguishing between causative and protective actions. Furthermore, obesity was linked with the 8-mer GGCGGAGT (χ^2^ = 9.49; p = 0.0021), whereby its 5-mer derivative GGAGT (block 4–8; χ^2^ = 10.96; p = 0.0009) and the 3-mer GGA (block 4–6; χ^2^ = 15.94; p = 0.00005) were the most significantly related (Table 5). Besides, several haplotypes were also implicated in MI. These included two 8-mers ATTGGGAC (χ^2^ = 6.68; p = 0.010) and GGTGGGAT (χ^2^ = 4.25; p = 0.039), which exhibited similar trends culminating in the 4-mer ATTG (χ^2^ = 13.92; p = 0.0002) being the most significantly involved. Notably, in this population, the 8-mer ATTGGGAC (χ^2^ = 6.68; p = 0.010) was associated in a causative fashion with MI, while the ATTAGGAC (χ^2^ = 9.99; p = 0.0016) was protective against the disease, properties which were shared with T2DM (AGT Haplo Additional file 2: Suppl data).

Most importantly, several haplotypes were shared among the various risk traits. For example, the 5-mer GGAGT posed risk for HTN (χ^2^ = 11.48; p = 0.0007), obesity (χ^2^ = 10.96; p = 0.0009), while the 3-mer AGT was also shared by HTN (χ^2^ = 13.77; p = 0.0002) and obesity (χ^2^ = 9.62; p = 0.0019). The greatest similarities were however found between T2DM and CAD/MI which shared not only causative and protective 8-mer haplotypes, but several other causative derivatives including TGGGAC, TTGGGAC and ATTG, just to name a few. Put together, several haplotypes, particularly those encompassing the rs699G > A, rs4762G > A and rs3789679G > A were shared among HTN, T2DM and obesity as well as with CAD/MI in the present study. The complete list of the haplotypes associated with each disease trait is provided as supplementary data (AGT Haplo Additional file 2: Suppl data).

## Discussion

In the present study, we tested the likelihood of *AGT* polymorphism interacting with important cardiovascular risk traits, such as HTN, T2DM and obesity, contributing to disease pathways leading to atherosclerosis, following our previous finding of its linkage to early onset of CAD in heterozygous familial hyperlipidaemia (unpublished results). Accordingly, we employed the eight SNPs investigated in the early onset CAD study in the same ethnically homogeneous, angiographed cohort of native Saudi individuals, in which each subset of the cardiovascular disease traits of interest was well-documented. Our observations point to the different *AGT* variants as invariably conferring risk for these traits in our study population, whereby the most significant relationships involved the HTN patient group. This disease was linked to six of the eight studied SNPs (rs2067853, rs7071, rs699, rs3789679, rs2148582 and rs5051) in a causative fashion, unequivocally demonstrating the impact of *AGT* polymorphism as a risk for acquiring HTN. Of these variants, the rs699 (p.268 T) exhibited by far the most significant relationship, in agreement with several other studies that have implicated it in this disease in other ethnic groups, including Caucasians[36], Chinese[8], Slovaks[37], Polish[17] and Tunisians[19], among others. Therefore, our study reaffirms its place as a risk variant for HTN globally. However, current literature does not seem to hold much data on the other SNPs implicated in HTN in the present study. Therefore, our observations with respect to their involvement in HTN probably point to the novelty of their role as risk factors for the disease, at least in native Saudis. On the other hand, the lack of association for the rs4762 (p.M207T) with HTN in the present study appears to be in conflict with those reports implicating it in the disease [8-10].

Interestingly, rather than being involved in HTN, our data indicates that the rs4762 is associated with obesity and MI in the Saudi general population. To our knowledge, apart from a report suggesting an association of the SNP with central obesity-related HTN in the Hong Kong Chinese [8], literature seems to be also lacking with respect to its potential role in metabolic disorders, in general. Besides, we also observed some sharing of causative properties for four of the variants by HTN and CAD/MI as well as one (rs3789679) by HTN and obesity, all of which similarly appear to be yet undocumented in the literature. Thus, put together, our study identified partly novel associations for various *AGT* variants concomitantly predisposing individuals to multiple cardiovascular disease traits, pointing to potential pleiotropic activity of the *AGT* polymorphism on the risk of acquiring atherosclerosis.

The primary question raised in this study was whether or not the interactions of the *AGT* gene with these risk traits might explain some of the disease pathways to atherosclerosis. To begin with our data suggested that although initially the studied SNPs appeared to be associated with CAD almost all association were abolished when adjusted for age. On the other hand, the relationships of the variants with the disease traits were retained in the CAD population, pointing to these relationships as being essentially independent of the presence or absence of CAD. Notably, however, T2DM, which exhibited no delineable relationship with any of the variants in the general population, was weakly linked to three of the variants, rs1926723, rs2148582 and rs5051, possibly suggestive of the potential requirement for CAD presence in their interactions with the disease. Furthermore, each disease subset seemed to display unique variants that were linked to CAD. However, some similarities between the T2DM and HTN groups were observed in the associations of the variants with CAD, despite the above-mentioned lack of association with the former in the general population. Moreover, different combinations of these traits also produced unique trends. Thereby, the rs699 and rs5051 appeared to be invariably implicated in the different traits. These results imply therefore that the impact of the changes in the *AGT* gene sequence on CAD may be partly dependent on the type of disease trait involved.

The role of *AGT* polymorphism, particularly the rs699 and rs4762, in CAD has also been a subject of intense research interest recently. In the present setting, while the rs699 showed a strong link, the rs4762 exhibited only borderline association with CAD. As is the case with HTN, our findings concur with the reports of the rs699 predisposing individuals to developing CAD/MI in a diversity of ethnic groups, including the Egyptian [13], Japanese [16] and Spanish [21] populations. However, the finding of a weak relationship for the rs4762 is not in complete agreement with those linking it to CAD [8,18,19,31]. Besides, both the p.207 T and p268T have not only been implicated in CAD, but also in the severity of atherosclerosis [24,25], requirement for coronary artery bypass surgery [38], and even as predictors of restenosis recurrence after angioplasty [39,40], suggesting a multi-faceted role for these two variants in CAD, as a whole. Our data also provides some support for an association of the *AGT* polymorphism with the severity of atherosclerosis, independently of other cardiovascular risk traits, possibly strengthening the hypothesis of its involvement in the extent of the disease reported elsewhere [24,25]. Apart from these two, we also established the sharing of four other variants by HTN and CAD. Since alterations in blood pressure constitute a complex risk trait for CAD, this sharing of causative variants by CAD and HTN, its important predisposing disease, in this and other ethnic populations seems to strongly implicate gene-disease interactions in pathways leading to CAD. A number of studies have produced evidence pointing to the importance of gene-environment and gene-disease interactions in complex diseases such as CAD and its predisposing traits^43-50^. Thus, our findings indeed furnish strong support for the notion of such *AGT* interactions with the cardiovascular risk traits as a triggering factor for CAD.

Nonetheless, it should be noted that various other studies involving different ethnic populations, including the Chinese [41,42], Indians [28] and Germans [43], failed to establish any contribution of these variants to the risk of CAD or HTN, possibly pointing to ethnic variability in the practical relevance of these findings globally. Several factors might be responsible for these discrepancies. One of the most likely explanations is the probability of inter-ethnical variation in the minor allele frequencies among the various ethnic groups. Thus for example, the frequency of the 268 T has been found to vary significantly between black and white subjects in some studies, leading to the suggestion of the 268TT genotype as an independent risk for coronary events selectively in black post-myocardial infarction patients [30]. Besides, some studies have also implicated gender and age in the association of these genetic changes with disease. In the present study, gender appeared not to impact the overall relationships for the different traits. There was also no delineable confounding effect of family history or smoking on the interactions of the *AGT* gene with disease in our population.

The partial lack of consistency in some of our findings on the individual variants with other previous observations raised our curiosity as to whether haplotyping might provide better insight into potential gene-disease interactions that may contribute to CAD pathways. To begin with, as observed with the individual variants, by far the greater majority of the haplotype constructs were associated with HTN, in a much more significant fashion than did the constituent variants themselves. In particular, it was noteworthy that the most common 8-mer GGTGGGGT itself was implicated in HTN. Besides, these associations culminated in the 5-mer or 4-mer derivatives encompassing the three variants rs699G > A, rs4762G > A and rs3789679G > A exhibiting the most conspicuous relationships. Similar trends were also observed for obesity, T2DM and MI, whereby each of the traits was associated with a couple of the 8-mer haplotypes and their shorter derivatives. Most importantly, T2DM shared several causative haplotypes with CAD/MI. Moreover, many other haplotypes were also common to two or more of the disorders, similarly pointing to the presence of the rs699 and rs3789679 as the core loci for these traits. Not only were the haplotypes more significantly involved, but they also included causative sequences common to the risk traits among themselves and partly shared with CAD. Hence, while the associations of the individual variants with the disease traits do not unequivocally delineate the indicators for their interactive involvement in atherosclerosis, haplotyping seems to reveal several characteristics which may help decipher such relationships. These observations indeed implicate haplotypes as more reliable indicators for the involvement of the *AGT* gene in the different diseases. Notably, the involvement of haplotypes in the gene-environmental interactions in CAD has also been highlighted recently, with some studies describing events such a female sex-related segregation with hypertension [32]. Therefore, our findings support the notion of complex interactions of such traits offering some basis for a mechanistic explanation for the events leading to CAD. The putative mechanisms involved in these interactions need however to be addressed further to shed light on this complex subject.

In this regard, it is noteworthy that three of the variants investigated herein, which are also integral constituents of the reported causative haplotypes, are non-coding SNPs, residing either upstream in the promoter region or downstream in the untranslated portion of the gene. This raises the crucial question as to the nature of their role on disease pathways. Some other *AGT* variants, in particular those residing in the promoter region of the gene, have also been associated with disease, similarly raising an interest in these sequences as key players in the mechanisms involved in its interactions with disease. Thus, since only changes involving the two coding variants, rs699 and rs4762, may be directly responsible for triggering alterations related to protein functional expression associated with disease, it can certainly be speculated that the mechanisms involving alterations at such loci are not directly ascribable to such changes in the AGT protein functional expression. Hence, the findings of associations for both coding and non-coding *AGT* variants with cardiovascular disease traits implicate various mechanisms possibly involving both protein functional changes and gene regulatory processes in CAD pathways. The potential role of the 3’UTR in disease is increasingly gaining acknowledgement, as it contains important elements, particularly the micro RNAs, regulating certain properties of the gene, such as mRNA maturation. While not much speculation can be derived from the current observations alone, it seems nonetheless reasonable to argue that such mechanisms might be related to gene regulatory processes. The findings also provide a working basis for the notion of the *AGT* 3’UTR-trait interactions as an important component of pathways leading to atherosclerosis. This notion thus advocates for further studies on this subject.

## Conclusions

We therefore conclude that the *AGT* constitutes an independent risk gene for HTN, obesity and MI, which may be important in the manifestation of CAD associated with these disorders. Together with the discovery of several common causative haplotypes for these cardiovascular risk traits and CAD, these findings reaffirm the potential pleiotropic role of the *AGT* on disease pathways leading to atherosclerosis, possibly involving gene-disease interactive mechanisms.

## Abbreviations

AGT: Angiotensinogen; BMI: Body-mass index; CAD: Coronary artery disease; FH: Family history; HBP: High blood pressure; HL: Hyperlipidaemia; hTG: Hypertriglyceridaemia; hLDL: High low density lipoprotein; HTN: Primary hypertension; OBS: Obesity; SNP: Single nucleotide polymorphism; T2DM: Type 2 diabetes mellitus

## Competing interest

The authors declare that they have no competing interests.

## Authors’ contributions

MN was responsible for overall running the TaqMan assays, PM was involved in running the Affymerix assays, designing probes, screening for gene mutations as well as participating in the write up of the manuscript, AIT performed part of the statistical analysis, SE performed part of the sequencing experiments, DG ran part of the TaqMan assays, EA was responsible for clinical patient data and material acquisition, NM was responsible for patient recruitment and sample collection, NA contributed to statistical analysis, MA supervised the recruitment of the patients and compliance with Institutional ethical procedures, ND is the Principal Investigator, with the overall responsibility for the project and preparation of the manuscript. All authors read and approved the final manuscript.

## Pre-publication history

The pre-publication history for this paper can be accessed here:

http://www.biomedcentral.com/1471-2261/13/17/prepub

## Supplementary Material

Additional file 1 Associastion of individual AGT markers with different Cardiovascular risk factor. Click here for file

Additional file 2 Statistical Analysis of the diseased traits versus Angiotensinogen variants. Click here for file
