# Supplementary material for: Association of the angiotensinogen gene polymorphism with atherosclerosis and its risk traits in the Saudi population
Source: BMC Cardiovasc Disord. 2013 Mar 11;13:17. doi: 10.1186/1471-2261-13-17 (PMC3605175; doi:10.1186/1471-2261-13-17)
Supplement: Additional file 2 — Statistical Analysis of the diseased traits versus Angiotensinogen variants. [file 1471-2261-13-17-S2.docx]

**Supplementary data**

| **Suppl data 1:** Important characteristic of the studied SNPs | | | | | | | | | |
| --- | --- | --- | --- | --- | --- | --- | --- | --- | --- |
|  | Gene Locus | db SNP ID | Variation | AA change | Chromosomal Loci | MAF | HapMap MAF | | |
|  |  |  |  |  |  |  | CEU | HCB | YRI |
| **1** | 3’-UTR | rs2067853 | C>T | n.a. | 1:230838258 | 0.23(G) | 0.19(A) | N/A | 0.07(A) |
| **2** | 3’-UTR | rs7079 | C>A | n.a. | 1:230838331 | 0.41(A) | 0.33(A) | 0.19(A) | 0.06(A) |
| **3** | Intronic | rs1926723 | A>G | n.a. | 1:230840096 | 0.00(G) | 0.11(G) | 0.34(G) | 0.01(G) |
| **4** | Exon 2 | rs699 | C>T | Met>Thr | 1:230845794 | 0.0(C ) | 0.41(C) | 0.28(T) | 0.08(T) |
| **5** | Exon 2 | rs4762 | C>T | Thr>Met | 1:230845977 | 0.0(C) | 0.11(T) | 0.08(T) | 0.04(T) |
| **6** | Intronic | rs3789679 | G>A | n.a. | 1:230849694 | 0.0(A) | 0.1(A) | 0.34(A) | 0.09(A) |
| **7** | Intronic | rs2148582 | C>T | n.a. | 1:230849799 | 0.0(T) | 0.4(C) | 0.29(T) | 0.08(T) |
| **8** | 5’-UTR | rs5051 | T>C | n.a. | 1:230849872 | 0.5 | 0.4(T) | 0.28(C) | 0.06(C) |
| The table shows the important properties of the studied SNPs and their minor allele frequency (MAF) distributions in our study family, European Caucasian (CEU), Chinese Han (HCB) and Yoruba (YRI) populations. AA, amino acid; Met, methionine; Thr, threonine 3’UTR, 3-prime untranslated region | | | | | | | | | |

| **Suppl data 2:** Important clinical characteristics of the studied family. | | | | | | |
| --- | --- | --- | --- | --- | --- | --- |
| **Candidate** | **Sex** | **Age** | **Chol** | **TG** | **LDL-C** | **HDL-C** |
| FT | M | 40 | 6.1* | 0.8 | 4.0 | 1.06 |
| MT | F | 40 | 6.3** | 0.5 | 4.2** | 1.04 |
| S1 | M | 20 | 5.3 | 0.4 | 3.6 | 0.86** |
| S2 | M | 17 | 3.1 | 0.2 | 1.5 | 1.35 |
| S3 | M | 14 | 10.1** | 0.3 | 7.9** | 0.51** |
| S4 | M | 12 | 6.2* | 1.0 | 4.4** | 0.98** |
| S5 | M | 10 | 4.7 | 0.6 | 2.6 | 1.36 |
| S6 | M | 9 | 6.9** | 1.0 | 4.6** | 1.05 |
| S7 | M | 3 | 3.3 | 0.5 | 1.4 | 1.61 |
| D1 | F | 15 | 4.8 | 0.8 | 3.2 | 0.67** |
| D2 | F | 5 | 6.2* | 0.7 | 4.3** | 1.00** |
| Ranges are: Total Cholesterol, total (Desirable: < 5.2 mmol/l; Borderline high: 5.2 - 6.2 mmol/l; High: ≥ 6.2 mmol/l); Triglycerides (Normal: < 1.7 mmol/l; Borderline high: 1.7 -2.25 mmol/l; High: 2.26 -5.64 mmol/l; Very high:≥ 5.65 mmol/l); LDL-cholesterol (Optimal: < 2.59 mmol/l; Low risk: 2.59 – 3.34 mmol/l; Borderline high: 3.37 – 4.12 mmol/l; High: 4.14 – 4.90 mmol/l; Very high: ≥ 4.92 mmol/l) and HDL-Cholesterol (Low HDL: <1.04 mmol/l; Normal: 1.04 – 1.55 mmol/l; Desirable: > 1.55 mmol/l); *Borderline high total cholesterol levels; **Out-of range levels. | | | | | | |

| **Suppl data 3:** Interaction of myocardial infarction with angiotensinogen variants (CAD as a dependent variable) | | | | | | |
| --- | --- | --- | --- | --- | --- | --- |
| Parameter | Beta | Std. Error | t | P-Value | 95% Confidence Interval | |
|  |  |  |  |  | Lower Bound | Upper Bound |
| Intercept | 1.255 | .395 | 3.174 | .002 | .480 | 2.030 |
| [MI=0] * rs2148582A | -1.429 | .627 | -2.279 | .023 | -2.659 | -.200 |
| [MI=1] * rs2148582A | .032 | .018 | 1.767 | .077 | -.003 | .067 |
| [MI=0] * rs2148582G | -1.448 | .626 | -2.313 | .021 | -2.675 | -.221 |
| [MI=1] * rs2148582G | 0^a^ | . | . | . | . | . |
| [MI=0] * rs5051C | .080 | .281 | .284 | .777 | -.470 | .630 |
| [MI=1] * rs5051C | -.026 | .018 | -1.485 | .138 | -.061 | .008 |
| [MI=0] * rs5051T | .104 | .280 | .372 | .710 | -.444 | .652 |
| [MI=1] * rs5051T | 0^a^ | . | . | . | . | . |
| [MI=0] * rs3789679A | .017 | .043 | .406 | .684 | -.067 | .101 |
| [MI=1] * rs3789679A | -.104 | .032 | -3.229 | .001 | -.168 | -.041 |
| [MI=0] * rs3789679G | 0^a^ | . | . | . | . | . |
| [MI=1] * rs3789679G | 0^a^ | . | . | . | . | . |
| [MI=0] * rs699A | .075 | .280 | .269 | .788 | -.473 | .623 |
| [MI=1] * rs699A | -.303 | .279 | -1.084 | .279 | -.851 | .245 |
| [MI=0] * rs699G | .087 | .279 | .312 | .755 | -.460 | .635 |
| [MI=1] * rs699G | -.288 | .279 | -1.030 | .303 | -.836 | .260 |
| [MI=0] * rs4762A | .097 | .281 | .345 | .730 | -.455 | .649 |
| [MI=1] * rs4762A | -.227 | .280 | -.811 | .417 | -.775 | .321 |
| [MI=0] * rs4762G | .089 | .280 | .317 | .751 | -.461 | .639 |
| [MI=1] * rs4762G | -.246 | .279 | -.881 | .379 | -.793 | .301 |
| [MI=0] * rs1926723C | -.043 | .045 | -.971 | .332 | -.131 | .044 |
| [MI=1] * rs1926723C | .092 | .035 | 2.597 | .009 | .022 | .161 |
| [MI=0] * rs1926723T | 0^a^ | . | . | . | . | . |
| [MI=1] * rs1926723T | 0^a^ | . | . | . | . | . |
| [MI=0] * rs2067853A | .010 | .020 | .485 | .628 | -.030 | .050 |
| [MI=1] * rs2067853A | -.026 | .015 | -1.797 | .072 | -.055 | .002 |
| [MI=0] * rs2067853G | 0^a^ | . | . | . | . | . |
| [MI=1] * rs2067853G | 0^a^ | . | . | . | . | . |
| [MI=0] * rs7079G | .008 | .015 | .528 | .598 | -.022 | .039 |
| [MI=1] * rs7079G | .027 | .012 | 2.345 | .019 | .004 | .050 |
| [MI=0] * rs7079T | 0^a^ | . | . | . | . | . |
| [MI=1] * rs7079T | 0^a^ | . | . | . | . | . |
| a. This parameter is set to zero because it is redundant. The table shows interactive relationships for the angiotensinogen SNPs with myocardial infarction on coronary artery disease. In the parameter column, 0 denotes controls and 1 denotes cases. Beta represents the statistical power of the test. MI, myocardial infarction; Std. Error, standard error; t, t-test. | | | | | | |

| **Suppl data 4.** Hypertension interaction with angiotensinogen SNPs (CAD as a dependent variable) | | | | | | |
| --- | --- | --- | --- | --- | --- | --- |
| Parameter | Beta | Std. Error | t | P-Value | 95% Confidence Interval | |
|  |  |  |  |  | Lower Bound | Upper Bound |
| Intercept | .371 | .549 | .675 | .500 | -.706 | 1.447 |
| [HTN=0] * rs2148582A | -.307 | .653 | -.470 | .638 | -1.587 | .973 |
| [HTN=1] * rs2148582A | .065 | .021 | 3.095 | .002 | .024 | .107 |
| [HTN=0] * rs2148582G | -.299 | .651 | -.459 | .646 | -1.576 | .977 |
| [HTN=1] * rs2148582G | 0^a^ | . | . | . | . | . |
| [HTN=0] * rs5051C | .009 | .036 | .249 | .804 | -.062 | .080 |
| [HTN=1] * rs5051C | .483 | .348 | 1.391 | .164 | -.198 | 1.165 |
| [HTN=0] * rs5051T | 0^a^ | . | . | . | . | . |
| [HTN=1] * rs5051T | .532 | .347 | 1.533 | .125 | -.148 | 1.213 |
| [HTN=0] * rs3789679A | -.055 | .077 | -.720 | .472 | -.205 | .095 |
| [HTN=1] * rs3789679A | -.074 | .035 | -2.099 | .036 | -.143 | -.005 |
| [HTN=0] * rs3789679G | 0^a^ | . | . | . | . | . |
| [HTN=1] * rs3789679G | 0^a^ | . | . | . | . | . |
| [HTN=0] * rs699A | -.041 | .025 | -1.660 | .097 | -.090 | .007 |
| [HTN=1] * rs699A | .035 | .246 | .143 | .886 | -.446 | .516 |
| [HTN=0] * rs699G | 0^a^ | . | . | . | . | . |
| [HTN=1] * rs699G | .064 | .245 | .261 | .794 | -.417 | .545 |
| [HTN=0] * rs4762A | .252 | .352 | .718 | .473 | -.437 | .942 |
| [HTN=1] * rs4762A | -.378 | .347 | -1.087 | .277 | -1.059 | .303 |
| [HTN=0] * rs4762G | .256 | .350 | .732 | .464 | -.430 | .943 |
| [HTN=1] * rs4762G | -.414 | .347 | -1.194 | .233 | -1.095 | .266 |
| [HTN=0] * rs1926723C | -.086 | .077 | -1.111 | .267 | -.237 | .066 |
| [HTN=1] * rs1926723C | .030 | .038 | .772 | .440 | -.046 | .105 |
| [HTN=0] * rs1926723T | 0^a^ | . | . | . | . | . |
| [HTN=1] * rs1926723T | 0^a^ | . | . | . | . | . |
| [HTN=0] * rs2067853A | -.005 | .030 | -.170 | .865 | -.063 | .053 |
| [HTN=1] * rs2067853A | -.017 | .017 | -.976 | .329 | -.050 | .017 |
| [HTN=0] * rs2067853G | 0^a^ | . | . | . | . | . |
| [HTN=1] * rs2067853G | 0^a^ | . | . | . | . | . |
| [HTN=0] * rs7079G | .038 | .023 | 1.638 | .101 | -.008 | .084 |
| [HTN=1] * rs7079G | .017 | .013 | 1.268 | .205 | -.009 | .043 |
| [HTN=0] * rs7079T | 0^a^ | . | . | . | . | . |
| [HTN=1] * rs7079T | 0^a^ | . | . | . | . | . |
| a. This parameter is set to zero because it is redundant. The table shows interactive relationships for the angiotensinogen SNPs with hypertension on coronary artery disease. In the parameter column, 0 denotes controls and 1 denotes cases. Beta represents the statistical power of the test. HTN, hypertension; Std. Error, t, t-test; standard error. | | | | | | |

| **Suppl data 5:** Obesity interaction with angiotensinogen SNPs (CAD as a dependent variable) | | | | | | |
| --- | --- | --- | --- | --- | --- | --- |
| Parameter | Beta | Std. Error | t | P-Value | 95% Confidence Interval | |
|  |  |  |  |  | Lower Bound | Upper Bound |
| Intercept | .008 | .354 | .024 | .981 | -.686 | .703 |
| [OBS=0] * rs2148582A | .482 | .501 | .963 | .336 | -.500 | 1.465 |
| [OBS=1] * rs2148582A | .022 | .032 | .710 | .478 | -.039 | .084 |
| [OBS=0] * rs2148582G | .429 | .501 | .856 | .392 | -.553 | 1.410 |
| [OBS=1] * rs2148582G | 0^a^ | . | . | . | . | . |
| [OBS=0] * rs5051C | -.030 | .025 | -1.234 | .217 | -.079 | .018 |
| [OBS=1] * rs5051C | .452 | .355 | 1.275 | .202 | -.243 | 1.148 |
| [OBS=0] * rs5051T | 0^a^ | . | . | . | . | . |
| [OBS=1] * rs5051T | .491 | .354 | 1.389 | .165 | -.202 | 1.185 |
| [OBS=0] * rs3789679A | -.010 | .047 | -.216 | .829 | -.102 | .082 |
| [OBS=1] * rs3789679A | -.087 | .052 | -1.663 | .096 | -.189 | .015 |
| [OBS=0] * rs3789679G | 0^a^ | . | . | . | . | . |
| [OBS=1] * rs3789679G | 0^a^ | . | . | . | . | . |
| [OBS=0] * rs699A | .024 | .250 | .098 | .922 | -.466 | .515 |
| [OBS=1] * rs699A | -.007 | .022 | -.300 | .764 | -.050 | .037 |
| [OBS=0] * rs699G | .071 | .250 | .285 | .776 | -.419 | .561 |
| [OBS=1] * rs699G | 0^a^ | . | . | . | . | . |
| [OBS=0] * rs4762A | .076 | .251 | .302 | .763 | -.416 | .567 |
| [OBS=1] * rs4762A | .010 | .031 | .316 | .752 | -.051 | .070 |
| [OBS=0] * rs4762G | .024 | .250 | .097 | .923 | -.465 | .514 |
| [OBS=1] * rs4762G | 0^a^ | . | . | . | . | . |
| [OBS=0] * rs1926723C | -.044 | .047 | -.933 | .351 | -.136 | .048 |
| [OBS=1] * rs1926723C | .052 | .060 | .859 | .390 | -.066 | .170 |
| [OBS=0] * rs1926723T | 0^a^ | . | . | . | . | . |
| [OBS=1] * rs1926723T | 0^a^ | . | . | . | . | . |
| [OBS=0] * rs2067853A | -.021 | .021 | -1.029 | .304 | -.062 | .019 |
| [OBS=1] * rs2067853A | -.018 | .026 | -.691 | .489 | -.068 | .033 |
| [OBS=0] * rs2067853G | 0^a^ | . | . | . | . | . |
| [OBS=1] * rs2067853G | 0^a^ | . | . | . | . | . |
| [OBS=0] * rs7079G | .023 | .016 | 1.412 | .158 | -.009 | .054 |
| [OBS=1] * rs7079G | .035 | .020 | 1.734 | .083 | -.005 | .074 |
| [OBS=0] * rs7079T | 0^a^ | . | . | . | . | . |
| [OBS=1] * rs7079T | 0^a^ | . | . | . | . | . |
| a. This parameter is set to zero because it is redundant. The table shows interactive relationships for the angiotensinogen SNPs with obesity (OBS) on coronary artery disease. In the parameter column, 0 denotes controls and 1 denotes cases. Beta represents the statistical power of the test. Std. Error; t, t-test; standard error. | | | | | | |

| **Suppl data 6:** Type 2 diabetes mellitus interaction with angiotensinogen SNPs (CAD a dependent variable). | | | | | | |
| --- | --- | --- | --- | --- | --- | --- |
| Parameter | Beta | Std. Error | t | P-Value | 95% Confidence Interval | |
|  |  |  |  |  | Lower Bound | Upper Bound |
| Intercept | -.089 | .418 | -.212 | .832 | -.908 | .731 |
| [DM=0] * rs2148582A | .617 | .483 | 1.276 | .202 | -.331 | 1.564 |
| [DM=1] * rs2148582A | .056 | .024 | 2.347 | .019 | .009 | .103 |
| [DM=0] * rs2148582G | .582 | .483 | 1.206 | .228 | -.364 | 1.529 |
| [DM=1] * rs2148582G | 0^a^ | . | . | . | . | . |
| [DM=0] * rs5051C | -.009 | .026 | -.336 | .737 | -.059 | .042 |
| [DM=1] * rs5051C | .522 | .342 | 1.529 | .126 | -.147 | 1.192 |
| [DM=0] * rs5051T | 0^a^ | . | . | . | . | . |
| [DM=1] * rs5051T | .592 | .341 | 1.738 | .082 | -.076 | 1.261 |
| [DM=0] * rs3789679A | .006 | .045 | .139 | .889 | -.081 | .094 |
| [DM=1] * rs3789679A | -.099 | .044 | -2.241 | .025 | -.186 | -.012 |
| [DM=0] * rs3789679G | 0^a^ | . | . | . | . | . |
| [DM=1] * rs3789679G | 0^a^ | . | . | . | . | . |
| [DM=0] * rs699A | -.051 | .019 | -2.715 | .007 | -.088 | -.014 |
| [DM=1] * rs699A | .102 | .241 | .421 | .673 | -.371 | .574 |
| [DM=0] * rs699G | 0^a^ | . | . | . | . | . |
| [DM=1] * rs699G | .123 | .241 | .511 | .609 | -.349 | .595 |
| [DM=0] * rs4762A | -.148 | .242 | -.612 | .541 | -.622 | .326 |
| [DM=1] * rs4762A | .038 | .022 | 1.732 | .083 | -.005 | .081 |
| [DM=0] * rs4762G | -.153 | .241 | -.634 | .526 | -.625 | .319 |
| [DM=1] * rs4762G | 0^a^ | . | . | . | . | . |
| [DM=0] * rs1926723C | -.085 | .050 | -1.709 | .087 | -.183 | .013 |
| [DM=1] * rs1926723C | .070 | .046 | 1.523 | .128 | -.020 | .159 |
| [DM=0] * rs1926723T | 0^a^ | . | . | . | . | . |
| [DM=1] * rs1926723T | 0^a^ | . | . | . | . | . |
| [DM=0] * rs2067853A | -.019 | .022 | -.867 | .386 | -.063 | .024 |
| [DM=1] * rs2067853A | -.006 | .019 | -.287 | .774 | -.043 | .032 |
| [DM=0] * rs2067853G | 0^a^ | . | . | . | . | . |
| [DM=1] * rs2067853G | 0^a^ | . | . | . | . | . |
| [DM=0] * rs7079G | .044 | .017 | 2.635 | .008 | .011 | .077 |
| [DM=1] * rs7079G | .015 | .015 | .978 | .328 | -.015 | .045 |
| [DM=0] * rs7079T | 0^a^ | . | . | . | . | . |
| [DM=1] * rs7079T | 0^a^ | . | . | . | . | . |
| a. This parameter is set to zero because it is redundant. The table shows interactive relationships for the angiotensinogen SNPs with type 2 diabetes mellitus (DM) on coronary artery disease. In the parameter column, 0 denotes controls and 1 denotes cases. Beta represents the statistical power of the test. Std. Error, standard error; t, t-test. | | | | | | |

| **Suppl data 7:** Sex interaction with angiotensinogen SNPs (CAD as a dependent variable) | | | | | | |
| --- | --- | --- | --- | --- | --- | --- |
| Parameter | Beta | Std. Error | t | P-Value | 95% Confidence Interval | |
|  |  |  |  |  | Lower Bound | Upper Bound |
| Intercept | -.342 | .487 | -.702 | .482 | -1.298 | .613 |
| [Sex=1] * rs2148582A | 1.281 | .645 | 1.987 | .047 | .017 | 2.545 |
| [Sex=2] * rs2148582A | .103 | .030 | 3.405 | .001 | .044 | .162 |
| [Sex=1] * rs2148582G | 1.256 | .645 | 1.948 | .051 | -.008 | 2.520 |
| [Sex=2] * rs2148582G | 0^a^ | . | . | . | . | . |
| [Sex=1] * rs5051C | -.012 | .022 | -.550 | .582 | -.055 | .031 |
| [Sex=2] * rs5051C | .275 | .346 | .796 | .426 | -.402 | .953 |
| [Sex=1] * rs5051T | 0^a^ | . | . | . | . | . |
| [Sex=2] * rs5051T | .342 | .345 | .993 | .321 | -.334 | 1.018 |
| [Sex=1] * rs3789679A | -.037 | .040 | -.922 | .357 | -.116 | .042 |
| [Sex=2] * rs3789679A | -.049 | .052 | -.951 | .342 | -.151 | .052 |
| [Sex=1] * rs3789679G | 0^a^ | . | . | . | . | . |
| [Sex=2] * rs3789679G | 0^a^ | . | . | . | . | . |
| [Sex=1] * rs699A | -.431 | .344 | -1.251 | .211 | -1.106 | .244 |
| [Sex=2] * rs699A | .287 | .345 | .833 | .405 | -.389 | .963 |
| [Sex=1] * rs699G | -.403 | .344 | -1.170 | .242 | -1.078 | .272 |
| [Sex=2] * rs699G | .336 | .344 | .977 | .328 | -.338 | 1.011 |
| [Sex=1] * rs4762A | .100 | .244 | .411 | .681 | -.378 | .579 |
| [Sex=2] * rs4762A | .006 | .030 | .202 | .840 | -.052 | .064 |
| [Sex=1] * rs4762G | .072 | .243 | .296 | .767 | -.405 | .549 |
| [Sex=2] * rs4762G | 0^a^ | . | . | . | . | . |
| [Sex=1] * rs1926723C | -.016 | .042 | -.375 | .708 | -.097 | .066 |
| [Sex=2] * rs1926723C | .030 | .059 | .508 | .611 | -.086 | .146 |
| [Sex=1] * rs1926723T | 0^a^ | . | . | . | . | . |
| [Sex=2] * rs1926723T | 0^a^ | . | . | . | . | . |
| [Sex=1] * rs2067853A | -.017 | .018 | -.932 | .351 | -.052 | .019 |
| [Sex=2] * rs2067853A | -.030 | .025 | -1.191 | .234 | -.079 | .019 |
| [Sex=1] * rs2067853G | 0^a^ | . | . | . | . | . |
| [Sex=2] * rs2067853G | 0^a^ | . | . | . | . | . |
| [Sex=1] * rs7079G | .020 | .014 | 1.413 | .158 | -.008 | .048 |
| [Sex=2] * rs7079G | .031 | .019 | 1.596 | .111 | -.007 | .068 |
| [Sex=1] * rs7079T | 0^a^ | . | . | . | . | . |
| [Sex=2] * rs7079T | 0^a^ | . | . | . | . | . |
| a. This parameter is set to zero because it is redundant. The table shows interactive relationships for the angiotensinogen SNPs with sex on coronary artery disease. In the parameter column, 0 denotes controls and 1 denotes cases. Beta represents the statistical power of the test. Std. Error, standard error; t, t-test. | | | | | | |

| **Suppl data 8:** Age interaction with angiotensinogen SNPs (CAD as a dependent variable) | | | | | | |
| --- | --- | --- | --- | --- | --- | --- |
| Parameter | Beta | Std. Error | t | P-Value | 95% Confidence Interval | |
|  |  |  |  |  | Lower Bound | Upper Bound |
| Intercept | .653 | .022 | 29.394 | .000 | .609 | .696 |
| [AgeUp=1] * rs2148582A | -.694 | .341 | -2.038 | .042 | -1.362 | -.026 |
| [AgeUp=2] * rs2148582A | -.640 | .339 | -1.890 | .059 | -1.305 | .024 |
| [AgeUp=3] * rs2148582A | -.132 | .476 | -.276 | .783 | -1.065 | .802 |
| [AgeUp=4] * rs2148582A | .393 | .338 | 1.161 | .245 | -.270 | 1.055 |
| [AgeUp=5] * rs2148582A | .014 | .043 | .336 | .737 | -.069 | .098 |
| [AgeUp=1] * rs2148582G | -.652 | .337 | -1.934 | .053 | -1.312 | .009 |
| [AgeUp=2] * rs2148582G | -.648 | .337 | -1.925 | .054 | -1.308 | .012 |
| [AgeUp=3] * rs2148582G | -.236 | .475 | -.496 | .620 | -1.167 | .696 |
| [AgeUp=4] * rs2148582G | .262 | .338 | .774 | .439 | -.402 | .925 |
| [AgeUp=5] * rs2148582G | 0^a^ | . | . | . | . | . |
| [AgeUp=1] * rs5051C | .010 | .036 | .273 | .785 | -.061 | .081 |
| [AgeUp=2] * rs5051C | .445 | .337 | 1.319 | .187 | -.216 | 1.106 |
| [AgeUp=3] * rs5051C | -.075 | .036 | -2.050 | .040 | -.146 | -.003 |
| [AgeUp=4] * rs5051C | -.119 | .040 | -2.948 | .003 | -.197 | -.040 |
| [AgeUp=5] * rs5051C | .006 | .042 | .145 | .885 | -.075 | .087 |
| [AgeUp=1] * rs5051T | 0^a^ | . | . | . | . | . |
| [AgeUp=2] * rs5051T | .468 | .335 | 1.396 | .163 | -.189 | 1.126 |
| [AgeUp=3] * rs5051T | 0^a^ | . | . | . | . | . |
| [AgeUp=4] * rs5051T | 0^a^ | . | . | . | . | . |
| [AgeUp=5] * rs5051T | 0^a^ | . | . | . | . | . |
| [AgeUp=1] * rs3789679A | .007 | .060 | .124 | .902 | -.111 | .126 |
| [AgeUp=2] * rs3789679A | -.142 | .068 | -2.068 | .039 | -.276 | -.007 |
| [AgeUp=3] * rs3789679A | -.138 | .064 | -2.167 | .030 | -.263 | -.013 |
| [AgeUp=4] * rs3789679A | -.020 | .085 | -.230 | .818 | -.186 | .147 |
| [AgeUp=5] * rs3789679A | .110 | .076 | 1.445 | .149 | -.039 | .259 |
| [AgeUp=1] * rs3789679G | 0^a^ | . | . | . | . | . |
| [AgeUp=2] * rs3789679G | 0^a^ | . | . | . | . | . |
| [AgeUp=3] * rs3789679G | 0^a^ | . | . | . | . | . |
| [AgeUp=4] * rs3789679G | 0^a^ | . | . | . | . | . |
| [AgeUp=5] * rs3789679G | 0^a^ | . | . | . | . | . |
| [AgeUp=1] * rs699A | -.018 | .028 | -.648 | .517 | -.073 | .037 |
| [AgeUp=2] * rs699A | .010 | .026 | .395 | .693 | -.040 | .060 |
| [AgeUp=3] * rs699A | .501 | .335 | 1.497 | .135 | -.155 | 1.158 |
| [AgeUp=4] * rs699A | -.368 | .337 | -1.090 | .276 | -1.029 | .294 |
| [AgeUp=5] * rs699A | -.046 | .028 | -1.632 | .103 | -.102 | .009 |
| [AgeUp=1] * rs699G | 0^a^ | . | . | . | . | . |
| [AgeUp=2] * rs699G | 0^a^ | . | . | . | . | . |
| [AgeUp=3] * rs699G | .540 | .335 | 1.614 | .107 | -.116 | 1.196 |
| [AgeUp=4] * rs699G | -.299 | .337 | -.887 | .375 | -.960 | .362 |
| [AgeUp=5] * rs699G | 0^a^ | . | . | . | . | . |
| [AgeUp=1] * rs4762A | .202 | .338 | .598 | .550 | -.461 | .865 |
| [AgeUp=2] * rs4762A | .003 | .037 | .071 | .944 | -.070 | .075 |
| [AgeUp=3] * rs4762A | -.406 | .336 | -1.206 | .228 | -1.065 | .254 |
| [AgeUp=4] * rs4762A | .081 | .038 | 2.160 | .031 | .008 | .155 |
| [AgeUp=5] * rs4762A | .049 | .036 | 1.356 | .175 | -.022 | .119 |
| [AgeUp=1] * rs4762G | .202 | .337 | .599 | .549 | -.458 | .861 |
| [AgeUp=2] * rs4762G | 0^a^ | . | . | . | . | . |
| [AgeUp=3] * rs4762G | -.381 | .335 | -1.138 | .255 | -1.038 | .276 |
| [AgeUp=4] * rs4762G | 0^a^ | . | . | . | . | . |
| [AgeUp=5] * rs4762G | 0^a^ | . | . | . | . | . |
| [AgeUp=1] * rs1926723C | .021 | .067 | .317 | .751 | -.110 | .152 |
| [AgeUp=2] * rs1926723C | -.043 | .073 | -.589 | .556 | -.186 | .100 |
| [AgeUp=3] * rs1926723C | .110 | .070 | 1.571 | .116 | -.027 | .248 |
| [AgeUp=4] * rs1926723C | -.073 | .086 | -.850 | .395 | -.241 | .095 |
| [AgeUp=5] * rs1926723C | -.018 | .080 | -.221 | .825 | -.174 | .139 |
| [AgeUp=1] * rs1926723T | 0^a^ | . | . | . | . | . |
| [AgeUp=2] * rs1926723T | 0^a^ | . | . | . | . | . |
| [AgeUp=3] * rs1926723T | 0^a^ | . | . | . | . | . |
| [AgeUp=4] * rs1926723T | 0^a^ | . | . | . | . | . |
| [AgeUp=5] * rs1926723T | 0^a^ | . | . | . | . | . |
| [AgeUp=1] * rs2067853A | .026 | .032 | .814 | .416 | -.037 | .089 |
| [AgeUp=2] * rs2067853A | -.065 | .031 | -2.106 | .035 | -.126 | -.004 |
| [AgeUp=3] * rs2067853A | -.020 | .031 | -.628 | .530 | -.081 | .042 |
| [AgeUp=4] * rs2067853A | .003 | .033 | .083 | .934 | -.061 | .067 |
| [AgeUp=5] * rs2067853A | -.002 | .032 | -.049 | .961 | -.065 | .062 |
| [AgeUp=1] * rs2067853G | 0^a^ | . | . | . | . | . |
| [AgeUp=2] * rs2067853G | 0^a^ | . | . | . | . | . |
| [AgeUp=3] * rs2067853G | 0^a^ | . | . | . | . | . |
| [AgeUp=4] * rs2067853G | 0^a^ | . | . | . | . | . |
| [AgeUp=5] * rs2067853G | 0^a^ | . | . | . | . | . |
| [AgeUp=1] * rs7079G | .017 | .024 | .718 | .473 | -.030 | .065 |
| [AgeUp=2] * rs7079G | .066 | .025 | 2.687 | .007 | .018 | .114 |
| [AgeUp=3] * rs7079G | .014 | .025 | .549 | .583 | -.035 | .062 |
| [AgeUp=4] * rs7079G | .030 | .026 | 1.158 | .247 | -.021 | .080 |
| [AgeUp=5] * rs7079G | .005 | .025 | .208 | .835 | -.044 | .054 |
| [AgeUp=1] * rs7079T | 0^a^ | . | . | . | . | . |
| [AgeUp=2] * rs7079T | 0^a^ | . | . | . | . | . |
| [AgeUp=3] * rs7079T | 0^a^ | . | . | . | . | . |
| [AgeUp=4] * rs7079T | 0^a^ | . | . | . | . | . |
| [AgeUp=5] * rs7079T | 0^a^ | . | . | . | . | . |
| a. This parameter is set to zero because it is redundant. The table shows interactive relationships for the angiotensinogen SNPs with age on coronary artery disease. Beta denotes the statistical power of the test. Std. Error, standard error; t, t-test; In the parameter column, AgeUp represents different cut-off points for the age groups, whereby 1 is up to 45 years; 2 is up to 50; 3 is up to 55; 4 is up to 60 and 5 means 65 or above. | | | | | | |

| **Suppl data 9**. Association of angiotensiogen variants with CAD following adjustment for age. | | | | | | | |
| --- | --- | --- | --- | --- | --- | --- | --- |
| Parameter | B | Std. Error | t | P-Value | Adjusted P-Value | 95% Confidence Interval | |
|  |  |  |  |  |  | Lower Bound | Upper Bound |
| Intercept | 0.653 | 0.022 | 29.39 | 0 |  | 0.609 | 0.696 |
| [AgeUp=1] * rs2148582A | -0.694 | 0.341 | -2.038 | 0.042 | 0.168 | -1.362 | -0.026 |
| [AgeUp=2] * rs2148582A | -0.64 | 0.339 | -1.89 | 0.059 | 0.295 | -1.305 | 0.024 |
| [AgeUp=3] * rs2148582A | -0.132 | 0.476 | -0.276 | 0.783 | 3.915 | -1.065 | 0.802 |
| [AgeUp=4] * rs2148582A | 0.393 | 0.338 | 1.161 | 0.245 | 1.225 | -0.27 | 1.055 |
| [AgeUp=5] * rs2148582A | 0.014 | 0.043 | 0.336 | 0.737 | 3.685 | -0.069 | 0.098 |
| [AgeUp=1] * rs2148582G | -0.652 | 0.337 | -1.934 | 0.053 | 0.265 | -1.312 | 0.009 |
| [AgeUp=2] * rs2148582G | -0.648 | 0.337 | -1.925 | 0.054 | 0.27 | -1.308 | 0.012 |
| [AgeUp=3] * rs2148582G | -0.236 | 0.475 | -0.496 | 0.62 | 3.1 | -1.167 | 0.696 |
| [AgeUp=4] * rs2148582G | 0.262 | 0.338 | 0.774 | 0.439 | 2.195 | -0.402 | 0.925 |
| [AgeUp=5] * rs2148582G | 0^a^ | . | . | . | #VALUE! | . | . |
| [AgeUp=1] * rs5051C | 0.01 | 0.036 | 0.273 | 0.785 | 3.925 | -0.061 | 0.081 |
| [AgeUp=2] * rs5051C | 0.445 | 0.337 | 1.319 | 0.187 | 0.935 | -0.216 | 1.106 |
| [AgeUp=3] * rs5051C | -0.075 | 0.036 | -2.05 | 0.04 | 0.2 | -0.146 | -0.003 |
| [AgeUp=4] * rs5051C | -0.119 | 0.04 | -2.948 | 0.003 | 0.015 | -0.197 | -0.04 |
| [AgeUp=5] * rs5051C | 0.006 | 0.042 | 0.145 | 0.885 | 4.425 | -0.075 | 0.087 |
| [AgeUp=1] * rs5051T | 0^a^ | . | . | . | #VALUE! | . | . |
| [AgeUp=2] * rs5051T | 0.468 | 0.335 | 1.396 | 0.163 | 0.815 | -0.189 | 1.126 |
| [AgeUp=3] * rs5051T | 0^a^ | . | . | . | #VALUE! | . | . |
| [AgeUp=4] * rs5051T | 0^a^ | . | . | . | #VALUE! | . | . |
| [AgeUp=5] * rs5051T | 0^a^ | . | . | . | #VALUE! | . | . |
| [AgeUp=1] * rs3789679A | 0.007 | 0.06 | 0.124 | 0.902 | 4.51 | -0.111 | 0.126 |
| [AgeUp=2] * rs3789679A | -0.142 | 0.068 | -2.068 | 0.039 | 0.195 | -0.276 | -0.007 |
| [AgeUp=3] * rs3789679A | -0.138 | 0.064 | -2.167 | 0.03 | 0.15 | -0.263 | -0.013 |
| [AgeUp=4] * rs3789679A | -0.02 | 0.085 | -0.23 | 0.818 | 4.09 | -0.186 | 0.147 |
| [AgeUp=5] * rs3789679A | 0.11 | 0.076 | 1.445 | 0.149 | 0.745 | -0.039 | 0.259 |
| [AgeUp=1] * rs3789679G | 0^a^ | . | . | . | #VALUE! | . | . |
| [AgeUp=2] * rs3789679G | 0^a^ | . | . | . | #VALUE! | . | . |
| [AgeUp=3] * rs3789679G | 0^a^ | . | . | . | #VALUE! | . | . |
| [AgeUp=4] * rs3789679G | 0^a^ | . | . | . | #VALUE! | . | . |
| [AgeUp=5] * rs3789679G | 0^a^ | . | . | . | #VALUE! | . | . |
| [AgeUp=1] * rs699A | -0.018 | 0.028 | -0.648 | 0.517 | 2.585 | -0.073 | 0.037 |
| [AgeUp=2] * rs699A | 0.01 | 0.026 | 0.395 | 0.693 | 3.465 | -0.04 | 0.06 |
| [AgeUp=3] * rs699A | 0.501 | 0.335 | 1.497 | 0.135 | 0.675 | -0.155 | 1.158 |
| [AgeUp=4] * rs699A | -0.368 | 0.337 | -1.09 | 0.276 | 1.38 | -1.029 | 0.294 |
| [AgeUp=5] * rs699A | -0.046 | 0.028 | -1.632 | 0.103 | 0.515 | -0.102 | 0.009 |
| [AgeUp=1] * rs699G | 0^a^ | . | . | . | #VALUE! | . | . |
| [AgeUp=2] * rs699G | 0^a^ | . | . | . | #VALUE! | . | . |
| [AgeUp=3] * rs699G | 0.54 | 0.335 | 1.614 | 0.107 | 0.535 | -0.116 | 1.196 |
| [AgeUp=4] * rs699G | -0.299 | 0.337 | -0.887 | 0.375 | 1.875 | -0.96 | 0.362 |
| [AgeUp=5] * rs699G | 0^a^ | . | . | . | #VALUE! | . | . |
| [AgeUp=1] * rs4762A | 0.202 | 0.338 | 0.598 | 0.55 | 2.75 | -0.461 | 0.865 |
| [AgeUp=2] * rs4762A | 0.003 | 0.037 | 0.071 | 0.944 | 4.72 | -0.07 | 0.075 |
| [AgeUp=3] * rs4762A | -0.406 | 0.336 | -1.206 | 0.228 | 1.14 | -1.065 | 0.254 |
| [AgeUp=4] * rs4762A | 0.081 | 0.038 | 2.16 | 0.031 | 0.155 | 0.008 | 0.155 |
| [AgeUp=5] * rs4762A | 0.049 | 0.036 | 1.356 | 0.175 | 0.875 | -0.022 | 0.119 |
| [AgeUp=1] * rs4762G | 0.202 | 0.337 | 0.599 | 0.549 | 2.745 | -0.458 | 0.861 |
| [AgeUp=2] * rs4762G | 0^a^ | . | . | . | #VALUE! | . | . |
| [AgeUp=3] * rs4762G | -0.381 | 0.335 | -1.138 | 0.255 | 1.275 | -1.038 | 0.276 |
| [AgeUp=4] * rs4762G | 0^a^ | . | . | . | #VALUE! | . | . |
| [AgeUp=5] * rs4762G | 0^a^ | . | . | . | #VALUE! | . | . |
| [AgeUp=1] * rs1926723C | 0.021 | 0.067 | 0.317 | 0.751 | 3.755 | -0.11 | 0.152 |
| [AgeUp=2] * rs1926723C | -0.043 | 0.073 | -0.589 | 0.556 | 2.78 | -0.186 | 0.1 |
| [AgeUp=3] * rs1926723C | 0.11 | 0.07 | 1.571 | 0.116 | 0.58 | -0.027 | 0.248 |
| [AgeUp=4] * rs1926723C | -0.073 | 0.086 | -0.85 | 0.395 | 1.975 | -0.241 | 0.095 |
| [AgeUp=5] * rs1926723C | -0.018 | 0.08 | -0.221 | 0.825 | 4.125 | -0.174 | 0.139 |
| [AgeUp=1] * rs1926723T | 0^a^ | . | . | . | #VALUE! | . | . |
| [AgeUp=2] * rs1926723T | 0^a^ | . | . | . | #VALUE! | . | . |
| [AgeUp=3] * rs1926723T | 0^a^ | . | . | . | #VALUE! | . | . |
| [AgeUp=4] * rs1926723T | 0^a^ | . | . | . | #VALUE! | . | . |
| [AgeUp=5] * rs1926723T | 0^a^ | . | . | . | #VALUE! | . | . |
| [AgeUp=1] * rs2067853A | 0.026 | 0.032 | 0.814 | 0.416 | 2.08 | -0.037 | 0.089 |
| [AgeUp=2] * rs2067853A | -0.065 | 0.031 | -2.106 | 0.035 | 0.175 | -0.126 | -0.004 |
| [AgeUp=3] * rs2067853A | -0.02 | 0.031 | -0.628 | 0.53 | 2.65 | -0.081 | 0.042 |
| [AgeUp=4] * rs2067853A | 0.003 | 0.033 | 0.083 | 0.934 | 4.67 | -0.061 | 0.067 |
| [AgeUp=5] * rs2067853A | -0.002 | 0.032 | -0.049 | 0.961 | 4.805 | -0.065 | 0.062 |
| [AgeUp=1] * rs2067853G | 0^a^ | . | . | . | #VALUE! | . | . |
| [AgeUp=2] * rs2067853G | 0^a^ | . | . | . | #VALUE! | . | . |
| [AgeUp=3] * rs2067853G | 0^a^ | . | . | . | #VALUE! | . | . |
| [AgeUp=4] * rs2067853G | 0^a^ | . | . | . | #VALUE! | . | . |
| [AgeUp=5] * rs2067853G | 0^a^ | . | . | . | #VALUE! | . | . |
| [AgeUp=1] * rs7079G | 0.017 | 0.024 | 0.718 | 0.473 | 2.365 | -0.03 | 0.065 |
| [AgeUp=2] * rs7079G | 0.066 | 0.025 | 2.687 | 0.007 | 0.035 | 0.018 | 0.114 |
| [AgeUp=3] * rs7079G | 0.014 | 0.025 | 0.549 | 0.583 | 2.915 | -0.035 | 0.062 |
| [AgeUp=4] * rs7079G | 0.03 | 0.026 | 1.158 | 0.247 | 1.235 | -0.021 | 0.08 |
| [AgeUp=5] * rs7079G | 0.005 | 0.025 | 0.208 | 0.835 | 4.175 | -0.044 | 0.054 |
| [AgeUp=1] * rs7079T | 0^a^ | . | . | . | #VALUE! | . | . |
| [AgeUp=2] * rs7079T | 0^a^ | . | . | . | #VALUE! | . | . |
| [AgeUp=3] * rs7079T | 0^a^ | . | . | . | #VALUE! | . | . |
| [AgeUp=4] * rs7079T | 0^a^ | . | . | . | #VALUE! | . | . |
| [AgeUp=5] * rs7079T | 0^a^ | . | . | . | #VALUE! | . | . |
| The table shows the influence of age on the association of the angiotensinogen gene with coronary artery disease. Bonferroni test was performed to correct for the effect of age on these relationships. | | | | | | | |
